# Professional identity and career planning in early-year medical students: the chain mediation of academic motivation and ability

**DOI:** 10.3389/fpsyg.2026.1821989

**Published:** 2026-09-10

**Authors:** Liang Zhou, Huibing Guo, Yibei Wang, Qiang He, Danmei Liang

**Affiliations:** 1 Department of Student Affairs Management, West China School of Medicine, West China Hospital, Sichuan University, Chengdu, China; 2 West China School of Medicine, Sichuan University, Chengdu, China

**Keywords:** academic ability, academic motivation, career planning, early-year medical students, professional identity

## Abstract

**Background:**

Career planning is important for early-year medical students, yet the factors associated with it remain incompletely understood. This study examined the association between professional identity and career planning, with particular attention to the mediating roles of academic motivation and academic ability.

**Methods:**

A cross-sectional study was conducted among 1,031 first- and second-year medical students from a Chinese university using cluster sampling. Self-reported measures assessed professional identity, academic motivation, academic ability, and career planning. Structural equation modeling was used to examine the proposed associations.

**Results:**

Career planning differed significantly across several demographic and educational-context characteristics. Professional identity was positively associated with career planning (β = 0.42, *p* < 0.001). Academic motivation and ability showed significant mediating effects, with a significant chain mediation effect through the sequential pathway from professional identity to academic motivation to academic ability to career planning [95% CI = (0.057, 0.107)].

**Conclusion:**

Professional identity was positively associated with career planning among early-year medical students, with significant mediating effects of academic motivation and ability. These findings suggest that professional identity, academic motivation, and academic ability may be relevant considerations in supporting early career development in medical education.

## 1 Introduction

Career planning refers to the process through which individuals explore career options, set goals, and formulate actionable strategies to achieve professional aspirations (Zhan, 2006). For medical students, career planning extends beyond deciding whether to become a physician; it also involves exploring possible professional pathways within medicine, setting realistic career goals, and preparing for those pathways. Career-development programs may improve students' satisfaction with career planning (Zink et al., 2007). However, factors associated with career planning during the early stages of medical education remain incompletely understood.

Professional identity refers to the representation of self that develops through the internalization of the characteristics, values, and norms of the medical profession (Cruess et al., 2019). Positive associations between professional identity and career-development outcomes have been reported in student and health-professions populations (Chen and Hung, 2022; Zhou et al., 2025), although evidence specifically concerning early-year medical students remains limited.

The early years of medical education represent a particularly relevant period for examining these associations for several reasons. First, students begin formal socialization into the medical profession after entering medical school, while their prior understanding of medical practice may still be relatively limited (Sarraf-Yazdi et al., 2021; Stubbing et al., 2018). Second, career preferences begin to develop and evolve during undergraduate medical training, making the early years an important period for initial career exploration (Querido et al., 2016). Third, professional identity is still actively developing during this stage of training (Jarvis-Selinger et al., 2012). Accordingly, examining professional identity and career planning during the first two years of medical school may help clarify how these constructs are related at an early stage of professional development.

Academic motivation, conceptualized as the psychological drive underlying goal-directed learning behaviors (Neufeld and Malin, 2020; Ryan and Deci, 2017), may statistically account for part of the association between professional identity and career planning. Among first-year medical students, professional identity and motivation for medical school have been positively associated (Faihs et al., 2023). In higher education, motivational factors have also been associated with career decidedness (Bargmann et al., 2022). These findings provide a rationale for examining the potential mediating role of academic motivation between professional identity and career planning.

In the present study, academic ability refers to self-perceived academic competence, intellectual growth, and competency development. It therefore does not represent objective academic performance such as GPA, examination scores, or clinical performance, and it is not assumed to be a necessary condition for career goal attainment. Previous research has reported associations between professional identity and academic self-efficacy or learning engagement in university students (Chen et al., 2024), while learning ability has also been associated with career planning in undergraduate students (Xue, 2024). Although these constructs and populations are not identical to those examined in the present study, they provide a rationale for testing academic ability as a second potential mediator between professional identity and career planning.

Furthermore, academic motivation and academic ability may also be related. Previous research has reported associations between motivational beliefs and subsequent academic achievement among medical students (Artino et al., 2010), and between motivation and academic performance (Kusurkar et al., 2013). Although academic performance is not equivalent to the self-perceived academic ability assessed in the present study, these findings provide a rationale for testing a potential sequential mediation pathway involving academic motivation and academic ability.

Building on this literature, we propose four hypotheses:

H1: Professional identity is positively associated with career planning among early-year medical students.

H2: Academic motivation mediates the association between professional identity and career planning.

H3: Academic ability mediates the association between professional identity and career planning.

H4: Academic motivation and ability sequentially mediate the association between professional identity and career planning (i.e., professional identity → academic motivation → academic ability → career planning).

The present study tests these hypotheses among early-year medical students in China. We also explore differences in career planning across selected demographic and educational-context characteristics. By incorporating professional identity, academic motivation, academic ability, and career planning within a single model, the study examines the individual and sequential mediating roles of academic motivation and academic ability during the early stage of medical education.

## 2 Materials and methods

### 2.1 Participants and procedures

This cross-sectional study was conducted in May 2024 at a university in Sichuan Province, China. The research team obtained approval from the university leadership and the Institutional Review Board (IRB No. 2024027) after presenting the study's objectives, design, and expected outcomes.

Using cluster sampling, we recruited all first- and second-year medical students (initial *N* = 1,078). Data collection took place in designated classrooms. The researchers and academic advisors were present to assist students in completing the questionnaires. Participants accessed the questionnaire platform (Wenjuanxing, https://www.wjx.cn/) by scanning a QR code. The first page of the questionnaire detailed the study's purpose, the voluntary nature of participation, and the confidentiality and anonymity of the data. Participants could start to fill it out only after clicking “Agree.” All questionnaire items were required for online submission, and no separate “no response” or “prefer not to answer” option was provided. However, participation was voluntary, and students could discontinue the questionnaire at any time without submitting their responses.

Upon completion of data collection, rigorous quality control procedures were applied to ensure data integrity. Questionnaires completed in less than 180 s were excluded. This threshold was established based on pilot testing with 30 students, which showed that the minimum time required for completion of all items was approximately 180 s. The average completion time in the final analytic sample was 296 s. Additionally, responses demonstrating clear patterns of careless responding were excluded based on two objective criteria: (1) contradictory responses to reverse-coded items within the same scale, and (2) straight-lining responses (identical answers across all items on a scale). These criteria are standard in survey research to identify inattentive responding (Meade and Craig, 2012). After applying these exclusion criteria, 1,031 questionnaires were retained for analysis, representing 95.64% of the 1,078 questionnaires initially obtained.

Demographic and educational-context variables were selected based on their potential relevance to career planning among medical and health-professions students. Major selection autonomy was included as an indicator of the degree of self-directed major choice. Residence (urban/rural) was included as a broad contextual characteristic. University satisfaction was measured by a single item asking students to rate their overall satisfaction with university life on a 5-point Likert scale and was included as a broad indicator of students' overall university experience (Jia and Wang, 2024). Daily recreation time was included to characterize students' allocation of time outside formal academic activities. Club participation was measured as the number of student organizations in which students participated, including academic/professional and interest-based organizations, and was categorized as 0, 1, 2, or 3 or more organizations. Academic failure was defined as having failed at least one required course examination since entering university.

### 2.2 Measures

*Professional Identity Scale for College Students (PISCS):* originally developed by Qin (2009), this 23-item instrument contains four dimensions: cognition (5 items), emotion (8 items), behavior (6 items), and adaptability (4 items). Responses were collected using a 5-point Likert scale (1 = “strongly disagree” to 5 = “strongly agree”), where higher total scores reflect stronger professional identity. The scale has demonstrated well-established reliability in medical students (Wu et al., 2022). In the current study, it demonstrated excellent reliability with a Cronbach's α of 0.95. The subscale α coefficients ranged from 0.92 to 0.96, indicating strong internal consistency for use with early-year medical students.

*Career Planning Scale (CPS):* Zhan's (2006) 30-item scale assesses six dimensions: self-cognition, career exploring, goal and plan, self-advancing, relationship, and feedback. Using a 4-point scale (1 = “never” to 4 = “always”), higher scores indicate clearer career planning. The scale has been validated among medical student populations (Guo et al., 2021). In this study, Cronbach's α = 0.94. The subscale α coefficients ranged from 0.89 to 0.95, indicating strong internal consistency across all dimensions for the target population.

*Academic Motivation Scale (AMS):* the scale was adapted from Long and Liu (2016) and consists of 7 items. Responses were recorded on a 5-point Likert scale (1 = “strongly disagree” to 5 = “strongly agree”), with higher scores denoting stronger academic motivation. Unlike multidimensional motivation scales, this tool measures the overall intensity of academic motivation, making it well-suited to the focus of this study. The scale's reliability and validity have been confirmed in a study involving medical students (Hu et al., 2024). In this study, Cronbach's α = 0.84.

*Academic Ability Scale (AAS):* developed by Feng et al. (2006), this 6-item scale assesses self-perceived academic competence, including three dimensions: skills enhancement, intellectual growth, and competency development. It employs a 5-point Likert scale (1 = “strongly disagree” to 5 = “strongly agree”), with total scores calculated by summation. The scale has been validated in multiple studies, demonstrating its effectiveness in evaluating university students' academic abilities, with confirmed reliability and validity (Yu et al., 2024; Zhong et al., 2016). In this study, it exhibited good reliability (Cronbach's α = 0.85), supporting its applicability for assessing self-perceived academic competence in early-year medical students.

### 2.3 Statistical analyses

T-tests and ANOVA were conducted to investigate demographic differences. Pearson correlation analysis evaluated the relationships between variables. Structural equation modeling (SEM) was utilized to examine chain mediation effects, with path analysis applied to the proposed model. Model fit indices included χ^2^/*df* < 5, CFI ≥ 0.90, TLI ≥ 0.90, NFI ≥ 0.90, RFI ≥ 0.90, IFI ≥ 0.90, RMSEA ≤ 0.08. To further evaluate the significance of parameter estimates, the bias-corrected nonparametric percentile Bootstrap method was implemented, utilizing 5,000 resamples to generate 95% confidence intervals. A mediation effect was deemed significant if the confidence interval excluded 0 (Preacher and Hayes, 2004). All statistical analyses were performed using SPSS 27.0 and AMOS 26.0.

## 3 Results

### 3.1 Demographics

The results of the demographic differences in career planning scores are presented in Table 1. The analysis showed that major selection autonomy was significantly associated with career-planning scores (*F* = 10.37, *p* < 0.001), with the highest scores observed among students who selected the major fully autonomously. Moreover, urban students had higher scores than rural students (*t* = 2.48, *p* = 0.013), whereas students with a history of academic failure had lower scores (*t* = −2.95, *p* = 0.003). Career-planning scores also differed significantly across levels of university satisfaction (*F* = 13.99, *p* < 0.001), daily recreation time (*F* = 22.55, *p* < 0.001), and club participation (*F* = 13.66, *p* < 0.001).

**Table 1:** Demographic differences in career planning.

Variables	N	M ±SD	Statistics, *p*
Residence			
Urban	741	78.18 ± 14.06	*t* = 2.48,
Rural	290	75.85 ± 12.17	*p* = 0.013
Academic failure			
Yes	157	74.59 ± 12.39	*t* = −2.95,
No	874	78.43 ± 12.28	*p* = 0.003
Major selection autonomy			
Fully autonomous decision	457	80.07 ± 14.64	*F* = 10.37,
Influenced by parental or external suggestions	526	76.10 ± 12.22	*p < * 0.001
Primarily determined by parents or others	38	70.66 ± 15.22	
Controlled by parental or external influence	10	72.70 ± 16.66	
University satisfaction			
Extremely satisfied	79	84.37 ± 15.06	*F* = 13.99,
Relatively satisfied	548	79.02 ± 13.42	*p < * 0.001
Moderate	359	74.46 ± 12.68	
Relatively dissatisfied	36	72.42 ± 11.31	
Extremely dissatisfied	9	69.33 ± 16.15	
Daily duration of recreation			
0–2 h	402	81.41 ± 14.33	*F* = 22.55,
3–5 h	510	75.60 ± 11.80	*p < * 0.001
5–7 h	79	74.70 ± 13.76	
>7 h	40	68.63 ± 16.46	
Number of clubs or organizations			
0	164	74.29 ± 14.09	*F* = 13.66,
1	330	75.67 ± 12.59	*p < * 0.001
2	343	78.20 ± 14.01	
3 or more	194	82.23 ± 12.74	

### 3.2 Correlation analysis

As shown in Table 2, professional identity was significantly and positively correlated with academic motivation (*r* = 0.60, *p* < 0.01), academic ability (*r* = 0.57, *p* < 0.01), and career planning (*r* = 0.59, *p* < 0.01). Additionally, significant positive correlations were observed between academic motivation, academic ability, and career planning, with significant correlations also evident across all measured dimensions (*p* < 0.01). These findings indicate that the study variables are interrelated, providing a basis for the subsequent mediation effect analysis.

**Table 2:** Correlation analysis of scores on each scale.

Variables	M	SD	1	2	3	4	5	6	7	8	9	10	11	12	13
1 Professional identity	87.12	13.6	-												
2 cognition	20.25	2.83	0.76^**^												
3 emotion	31.1	5.84	0.89^**^	0.60^**^											
4 behavior	22.12	4.25	0.86^**^	0.57^**^	0.63^**^										
5 adaptability	13.64	3.07	0.83^**^	0.53^**^	0.63^**^	0.72^**^									
6 Academic motivation	26.89	5.29	0.60^**^	0.40^**^	0.42^**^	0.68^**^	0.57^**^								
7 Academic ability	22.61	3.4	0.57^**^	0.44^**^	0.43^**^	0.54^**^	0.55^**^	0.66^**^							
8 Career planning	77.52	13.59	0.59^**^	0.49^**^	0.42^**^	0.59^**^	0.56^**^	0.55^**^	0.59^**^						
9 self-cognition	14.72	2.67	0.48^**^	0.42^**^	0.38^**^	0.44^**^	0.43^**^	0.41^**^	0.47^**^	0.83^**^					
10 career exploring	13.38	2.9	0.50^**^	0.41^**^	0.36^**^	0.48^**^	0.46^**^	0.37^**^	0.40^**^	0.77^**^	0.64^**^				
11 goal and plan	13.01	3.27	0.48^**^	0.36^**^	0.34^**^	0.48^**^	0.50^**^	0.51^**^	0.50^**^	0.83^**^	0.60^**^	0.56^**^			
12 self-advancing	14.51	2.72	0.57^**^	0.47^**^	0.40^**^	0.59^**^	0.52^**^	0.59^**^	0.59^**^	0.87^**^	0.69^**^	0.56^**^	0.70^**^		
13 relationship	13.71	3.32	0.50^**^	0.42^**^	0.33^**^	0.52^**^	0.49^**^	0.49^**^	0.52^**^	0.86^**^	0.63^**^	0.58^**^	0.63^**^	0.75^**^	
14 feedback	8.2	1.83	0.28^**^	0.24^**^	0.20^**^	0.27^**^	0.29^**^	0.23^**^	0.37^**^	0.64^**^	0.44^**^	0.36^**^	0.47^**^	0.50^**^	0.54^**^

^**^p < 0.01.

### 3.3 Mediation effect analysis

**Figure 1:**
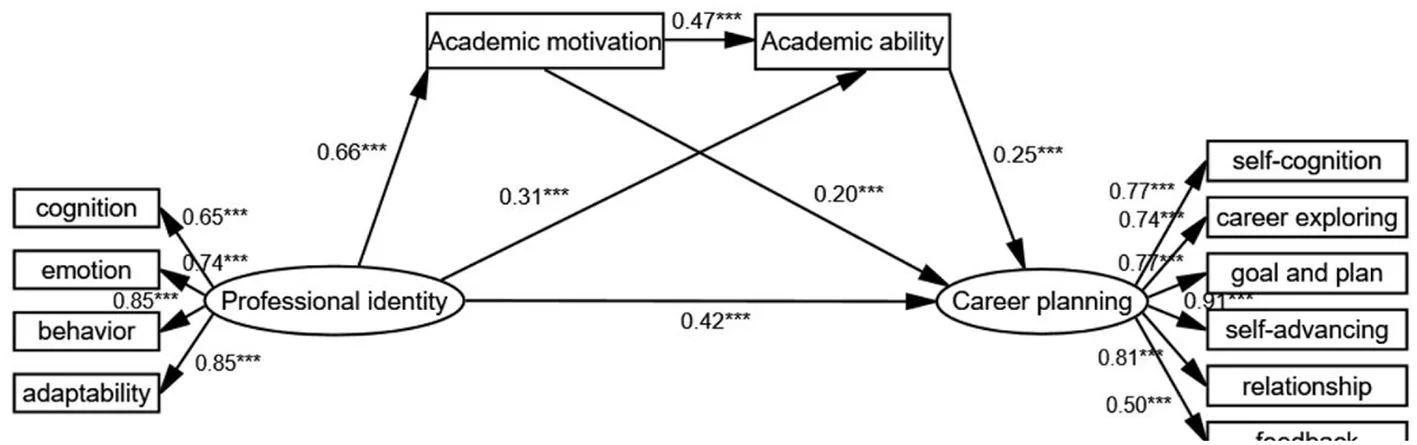
Chain mediation model of academic motivation and ability in the relationship between professional identity and career planning.

The mediation effect model and the path relationships between the variables are presented in Figure 1. SEM showed good fit indices: χ^2^/*df* = 3.97, NFI = 0.981, RFI = 0.966, IFI = 0.986, TLI = 0.974, CFI = 0.986, and RMSEA = 0.054. Professional identity was significantly and positively associated with career planning (β = 0.42, *p* < 0.001), academic motivation (β = 0.66, *p* < 0.001), and academic ability (β = 0.31, *p* < 0.001). Both academic motivation and academic ability were positively associated with career planning (β_1_ = 0.20, β_2_ = 0.25, *p* < 0.001). Academic motivation was also positively associated with academic ability (β = 0.47, *p* < 0.001).

Table 3 shows the significance of the chain mediation. The mediating effects of academic motivation [*b* = 0.13, 95% CI = (0.068, 0.173)] and academic ability [*b* = 0.08, 95% CI = (0.052, 0.104)] between professional identity and career planning were significant. Furthermore, a significant chain mediation effect was observed through the sequential pathway ‘professional identity → academic motivation → academic ability → career planning' [*b* = 0.08, 95% CI (0.057, 0.107)], explaining 12% additional variance.

**Table 3:** Chain mediating effects among professional identity, academic motivation, academic ability, and career planning.

Path	Effect	95% *CI*
Professional identity → Academic motivation → Career planning	0.13	[0.068, 0.173]
Professional identity → Academic ability → Career planning	0.08	[0.052, 0.104]
Professional identity → Academic motivation → Academic ability → Career planning	0.08	[0.057, 0.107]
Professional identity → Career planning (Direct effect)	0.42	[0.383, 0.486]
Total mediating effect	0.29	
Total effect	0.71	

## 4 Discussion

### 4.1 Demographics

This study identified notable variations in career planning across major selection autonomy. Students who made fully autonomous decisions had the highest career-planning scores (Silver, 2024). Additionally, less recreation time and greater club participation were associated with clearer career planning. The former may reflect more time allocated to academic and career exploration, while the latter is consistent with prior research on extracurricular engagement and career development (Ribeiro et al., 2024). However, the association between shorter recreation time and better career planning should be interpreted cautiously, as reduced leisure time may reflect greater academic engagement but may also indicate insufficient rest and poorer work–life balance.

### 4.2 The relationship between professional identity and career planning

This study confirmed that professional identity is significantly associated with career planning among early-year medical students, supporting Hypothesis 1 and aligning with Chen and Hung (2022). For these students, who have had limited clinical exposure, stronger professional identity was associated with clearer career planning and greater career exploration. This early stage may therefore represent a relevant period for career-development support.

These findings have several practical implications for medical education. First, institutions should prioritize fostering professional identity in early-year students, given its positive association with career planning. This may include structured approaches such as introductory courses that help students explore career options and understand the profession. Second, supporting academic motivation and competence development is also important. Mentorship programs with faculty or senior peers can provide tailored guidance and support students' academic and career development (Balandya et al., 2022). Finally, early clinical and research exposure may help students gain a better understanding of the profession and clarify their career goals. Because the present study did not evaluate these educational approaches, their effectiveness should be examined prospectively.

### 4.3 The mediating roles of academic motivation and ability

Academic motivation showed a significant mediating effect between professional identity and career planning, supporting Hypothesis 2. Professional identity and motivation have previously been positively associated among first-year medical students (Faihs et al., 2023), while motivational factors have also been associated with career decidedness among higher-education students (Bargmann et al., 2022). The present findings extend these associations by supporting the mediating role of academic motivation in the association between professional identity and career planning in this sample.

Academic ability also showed a significant mediating effect between professional identity and career planning, supporting Hypothesis 3. In the present model, students reporting stronger professional identity also tended to report greater academic ability, which was in turn associated with clearer career planning. Previous research has reported associations between professional identity and academic self-efficacy or learning engagement (Chen et al., 2024), while learning ability has been associated with career planning among undergraduate students (Xue, 2024). Although these studies involved different student populations and related but not identical constructs, they provide contextual support for examining the mediating role of academic ability. A possible explanation is that students with a stronger professional identity may internalize the values and expectations of the medical profession more deeply, which could enhance their confidence in mastering academic competencies and thereby promote more proactive career planning.

The chain mediation effect through academic motivation and ability was also significant, supporting Hypothesis 4. These findings are consistent with a sequential process in which professional identity may energize academic motivation, which in turn fosters perceived academic competence, and ultimately contributes to clearer career planning. From a developmental perspective, this chain is particularly meaningful for early-year medical students: a strong professional identity may provide the initial motivational drive to engage in academic work; as students perceive themselves becoming more competent through this engagement, their confidence in navigating future career paths is reinforced. Notably, the chain mediation effect explained an additional 12% of the variance in career planning beyond the separate mediating effects, indicating that the joint operation of motivation and ability captures a unique aspect of the career-planning process.

By incorporating academic motivation and ability simultaneously, the present model identifies two significant specific mediating effects and a significant chain mediation effect. The findings suggest that academic motivation and ability may operate jointly in the association between professional identity and career planning. Importantly, by focusing on first- and second-year medical students, this study examines these relationships during an early stage of professional socialization, when academic development and career exploration are occurring concurrently. Academic motivation and academic ability are unlikely to be the only possible mediators; contextual factors such as advising, mentorship, curriculum, and institutional experiences may also serve as additional or alternative mediators and warrant examination in future research.

### 4.4 Limitations

This study has several limitations. First, this study was conducted at a single university and only included early-year medical students, which may limit the generalizability of the findings. The sample is not representative of all medical students across different institutions, regions, or training stages. Future research could include students from multiple institutions and different academic years to improve external validity. Second, all variables were assessed using self-report instruments, which may introduce common method bias and social desirability bias. Future research could employ multi-source data (e.g., combining self-reports with objective academic records) or qualitative methods, such as semi-structured interviews, to provide deeper insights into students' perspectives. Finally, the cross-sectional design does not establish temporal ordering or causal direction among professional identity, academic motivation, academic ability, and career planning. Longitudinal studies are therefore recommended to examine how these relationships develop across different stages of medical training and whether the temporal ordering proposed in the statistical model is supported.

## 5 Conclusion

This study found that professional identity was positively associated with career planning among early-year medical students. Significant mediating effects of academic motivation and perceived academic ability were also observed, including a significant chain mediation effect involving both variables. These findings provide an integrated view of how professional identity, academic motivation, and academic ability are related to career planning during the early stage of medical education. Medical schools may consider these factors when developing career-development support for early-year students. Future multicenter and longitudinal studies are needed to determine the temporal ordering of these relationships and to evaluate the effectiveness of related educational strategies.

## Data Availability

The raw data supporting the conclusions of this article will be made available by the authors, without undue reservation.

## References

[B1] Artino A. R. La Rochelle J. S. Durning S. J. (2010). Second-year medical students' motivational beliefs, emotions, and achievement. Med. Educ. 44, 1203–1212. doi: 10.1111/j.1365-2923.2010.03712.x 21091760

[B2] Balandya E. Sunguya B. Kidenya B. Nyamhanga T. Minja I. K. Mahande M. . (2022). Joint research mentoring through the community of young research peers: a case for a unifying model for research mentorship at higher learning institutions. Adv. Med. Educ. Pract. 13, 355–367. doi: 10.2147/AMEP.S356678 35478975 PMC9038151

[B3] Bargmann C. Thiele L. Kauffeld S. (2022). Motivation matters: predicting students' career decidedness and intention to drop out after the first year in higher education. High. Educ. 83, 845–861. doi: 10.1007/s10734-021-00707-6

[B4] Chen C. C. Hung C. H. (2022). Plan and then act: the moderated moderation effects of profession identity and action control for students at arts universities during the career development process. Healthcare. 10:1938. doi: 10.3390/healthcare10101938 36292385 PMC9601481

[B5] Chen Q. Zhang Q. Yu F. Hou B. (2024). Investigating structural relationships between professional identity, learning engagement, academic self-efficacy, and university support: evidence from tourism students in China. Behav. Sci. 14:26. doi: 10.3390/bs14010026 38247678 PMC10813133

[B6] Cruess S. R. Cruess R. L. Steinert Y. (2019). Supporting the development of a professional identity: general principles. Med. Teach. 41, 641–649. doi: 10.1080/0142159X.2018.1536260 30739517

[B7] Faihs V. Heininger S. McLennan S. Gartmeier M. Berberat P. O. Wijnen-Meijer M. (2023). Professional identity and motivation for medical school in first-year medical students: a cross-sectional study. Med. Sci. Educ. 33, 431–441. doi: 10.1007/s40670-023-01754-7 37261015 PMC10226964

[B8] Feng T. Su T. Hu X. Li H. (2006). The development of a test about learning adjustment of undergraduates. Acta Psychol. Sin. 38, 762–769.

[B9] Guo P. P. Fang Q. Du W. Chen L. (2021). Correlation analysis between self-leadership and career planning of nursing interns. Evid. Based Nurs. 7, 2387–2392.

[B10] Hu F. F. Huang M. L. Qu G. J. (2024). On the interrelationships and influencing mechanisms among medical students' learning motivation, professional calling, and learning burnout. Heilongjiang Educ. 31–34.

[B11] Jarvis-Selinger S. Pratt D. D. Regehr G. (2012). Competency is not enough: integrating identity formation into the medical education discourse. Acad. Med. 87, 1185–1190. doi: 10.1097/ACM.0b013e3182604968 22836834

[B12] Jia L. Wang X. (2024). Self-efficacy and life satisfaction mediate the relationship between perceived social support and career exploration among college students: a cross-sectional study. J. Psychol. 158, 368–382. doi: 10.1080/00223980.2024.2312870 38358782

[B13] Kusurkar R. A. ten Cate T. J. Vos C. M. P. Westers P. Croiset G. (2013). How motivation affects academic performance: a structural equation modelling analysis. Adv. Health Sci. Educ. 18, 57–69. doi: 10.1007/s10459-012-9354-3 22354335 PMC3569579

[B14] Long C. Z. Liu Z. M. (2016). The influence of learning motivation on autonomous learning behavior: the mediating role of learning ability. Chin. J. Appl. Psychol. 22, 203–210.

[B15] Meade A. W. Craig S. B. (2012). Identifying careless responses in survey data. Psychol. Methods 17, 437–455. doi: 10.1037/a0028085 22506584

[B16] Neufeld A. Malin G. (2020). How medical students' perceptions of instructor autonomy-support mediate their motivation and psychological well-being. Med. Teach. 42, 650–656. doi: 10.1080/0142159X.2020.1726308 32074464

[B17] Preacher K. J. Hayes A. F. (2004). SPSS and SAS procedures for estimating indirect effects in simple mediation models. Behav. Res. Methods Instrum. Comput. 36, 717–731. doi: 10.3758/BF03206553 15641418

[B18] Qin P. B. (2009). Characteristics and related research on professional identity of college students (master's thesis). Southwest University, Chongqing.

[B19] Querido S. J. Vergouw D. Wigersma L. Batenburg R. S. De Rond M. E. J. Ten Cate O. T. J. (2016). Dynamics of career choice among students in undergraduate medical courses. a BEME systematic review: BEME Guide No. 33. Med. Teach. 38, 18–29. doi: 10.3109/0142159X.2015.1074990 26372112

[B20] Ribeiro N. Malafaia C. Neves T. Menezes I. (2024). The impact of extracurricular activities on university students' academic success and employability. Eur. J. High. Educ. 14, 389–409. doi: 10.1080/21568235.2023.2202874

[B21] Ryan R. M. Deci E. L. (2017). Self-Determination Theory: Basic Psychological Needs In Motivation Development And Wellness, New York, NY: The Guilford Press. doi: 10.1521/978.14625/28806

[B22] Sarraf-Yazdi S. Teo N. How A. E. H. Lee C. K. (2021). A scoping review of professional identity formation in undergraduate medical education. J. Gen. Intern. Med. 36, 3511–3521. doi: 10.1007/s11606-021-07024-9 34406582 PMC8606368

[B23] Silver B. R. (2024). Major Transitions: how college students interpret the process of changing fields of study. High Educ. 87, 1027–1042. doi: 10.1007/s10734-023-01050-8 37362756 PMC10169191

[B24] Stubbing E. Helmich E. Cleland J. (2018). Authoring the identity of learner before doctor in the figured world of medical school. Perspect. Med. Educ. 7, 40–46. doi: 10.1007/S40037-017-0399-0 29305820 PMC5807265

[B25] Wu X. N. Yang S. H. Sun X. M. Li J. (2022). Relationship between personality portraits and professional identity: a latent profile analysis. J. Nurs. 29, 1–5.

[B26] Xue H. (2024). A study of life skills, learning abilities, and career planning of Chinese college students. Int. J. Res. Stud. Lang. Learn. 10, 67–74. doi: 10.5861/ijrsll.2024.022

[B27] Yu M. Q. Lu J. M. Zhang J. J. Wang H. (2024). The impact of problematic mobile network use on academic adaptation of college freshmen: a moderated mediation model. Chin. J. Health Psychol. 32, 1914–1920.

[B28] Zhan F. S. (2006). Research on career planning of college students (master's thesis). Qufu Normal University, Qufu.

[B29] Zhong Y. Q. Gao Y. X. Lu Q. Y. (2016). Relationships between social support, learning adaptation and academic performance of college freshmen. Chin. J. Health Educ. 32, 226–229.

[B30] Zhou W. Wu X. Yang Y. (2025). Educational disparities in professional identity and career path planning for newly graduated nurses in China. J. Contin. Educ. Nurs. 56, 158–164. doi: 10.3928/00220124-20250313-01 40167195

[B31] Zink B. J. Hammoud M. M. Middleton E. Moroney D. Schigelone A. (2007). A comprehensive medical student career development program improves medical student satisfaction with career planning. Teach. Learn. Med. 19, 55–60 doi: 10.1080/10401330709336624 17331000

